# Designing evaluation studies to optimally inform policy: what factors do policy-makers in China consider when making resource allocation decisions on healthcare worker training programmes?

**DOI:** 10.1186/s12961-018-0292-2

**Published:** 2018-02-23

**Authors:** Shishi Wu, Helena Legido-Quigley, Julia Spencer, Richard James Coker, Mishal Sameer Khan

**Affiliations:** 1 Saw Swee Hock School of Public Health, National University of Singapore, 12 Science Drive 2 #10-01, Singapore, 117549 Singapore; 2 Communicable Diseases Policy Research Group, London School of Hygiene & Tropical Medicine, Keppel St, London, WC1E 7HT United Kingdom; 3 Faculty of Public Health, Mahidol University, Bangkok, Thailand

**Keywords:** Evaluation, Framework, Informing policy decisions, Healthcare provider training

## Abstract

**Background:**

In light of the gap in evidence to inform future resource allocation decisions about healthcare provider (HCP) training in low- and middle-income countries (LMICs), and the considerable donor investments being made towards training interventions, evaluation studies that are optimally designed to inform local policy-makers are needed. The aim of our study is to understand what features of HCP training evaluation studies are important for decision-making by policy-makers in LMICs. We investigate the extent to which evaluations based on the widely used Kirkpatrick model – focusing on direct outcomes of training, namely reaction of trainees, learning, behaviour change and improvements in programmatic health indicators – align with policy-makers’ evidence needs for resource allocation decisions. We use China as a case study where resource allocation decisions about potential scale-up (using domestic funding) are being made about an externally funded pilot HCP training programme.

**Methods:**

Qualitative data were collected from high-level officials involved in resource allocation at the national and provincial level in China through ten face-to-face, in-depth interviews and two focus group discussions consisting of ten participants each. Data were analysed manually using an interpretive thematic analysis approach.

**Results:**

Our study indicates that Chinese officials not only consider information about the direct outcomes of a training programme, as captured in the Kirkpatrick model, but also need information on the resources required to implement the training, the wider or indirect impacts of training, and the sustainability and scalability to other settings within the country. In addition to considering findings presented in evaluation studies, we found that Chinese policy-makers pay close attention to whether the evaluations were robust and to the composition of the evaluation team.

**Conclusions:**

Our qualitative study indicates that training programme evaluations that focus narrowly on direct training outcomes may not provide sufficient information for policy-makers to make decisions on future training programmes. Based on our findings, we have developed an evidence-based framework, which incorporates but expands beyond the Kirkpatrick model, to provide conceptual and practical guidance that aids in the design of training programme evaluations better suited to meet the information needs of policy-makers and to inform policy decisions.

## Background

Decisions on how best to allocate limited resources to improve health are often challenging for policy-makers in low- and middle-income countries (LMICs) because empirical evidence from studies conducted in their contexts is insufficient, and studies that are conducted do not provide information on factors that are critical to policy-makers in a format that is accessible to them [1–4]. Studies have indicated that inappropriate, overly complex presentation of research findings, absence of clear recommendations, low policy-relevance of research topics addressed and inadequate technical capacity of policy-makers to translate research findings into policy limit the utilisation of evidence by policy-makers [2, 5–8]. Further, a lack of timeliness in presenting research findings, few formal communication channels and mutual mistrust between policy-makers and researchers were also identified as barriers in two systematic reviews [9, 10]. As a result, studies have shown that health policy-makers often primarily rely on experience, values and subjective emotional reactions when making decisions, with less consideration given to evidence from research studies [11–14].

Barriers to applying evidence from evaluation studies to inform resource allocation decisions on strengthening health-related human resource capacity are particularly salient at present, as training interventions have received substantial attention and investment owing to the acute shortage of skilled healthcare providers (HCPs) in LMICs [15–18]. For example, between 2002 and 2010, the Global Fund to Fight AIDS, tuberculosis (TB) and malaria – the largest non-governmental funder of human resources – invested US$ 1.3 billion in human resource development activities, and it is estimated that more than half of this budget was invested in disease-focused training activities [19]. However, a recent systematic review found a very limited number of evaluation studies on HCP training in HIV, TB and malaria control programmes globally [20], leaving external donors and national policy-makers without essential information to base decisions about improvements to existing training programmes and possible scale-up or discontinuation.

Recognising the major evidence gap on the impact of HCP training and the considerable investments being made towards it, WHO has developed a guide to aid evaluations based on the Kirkpatrick model [15, 21]. The Kirkpatrick model, which identifies four levels of training outcomes that need to be evaluated, namely reaction, learning, behaviour and results [22], was originally designed in the 1950s to guide training evaluations in business and industry and forms the basis of various updated frameworks developed subsequently [23–28]. Even though a wide range of tools and frameworks facilitating evaluation of training programmes have been developed in recent decades [22, 23, 26–32], it remains the most widely applied.

Despite its popularity among evaluators and trainers, researchers have identified several limitations of the Kirkpatrick model, such as its simple assumption of causality and the implication that results and behaviour are more important than learning and reaction in assessing impact [33]. Since health policy formulation is influenced by diverse and complex considerations [2, 6, 34], we hypothesise that evaluations based on the Kirkpatrick model – focusing on the assessment of four direct training outcomes without providing information about broader factors that policy-makers consider – may result in evaluations that are too narrow in scope to optimally inform policy decisions [35]. Bowen and Zwi outline six policy-making models that help contextualise the translation of evidence into health policy [36]. The ‘Interactive Model’ takes as a starting point the complexity of the policy-making process and suggests that the search for evidence expands beyond research to include a number of other sources such as politics and interests [36]. This conceptualisation of the evidence-policy nexus differs from more linear models of policy-making that assume there is a direct relationship between knowledge generation and policy formulation. It further highlights the need for research to be more ‘fit for purpose’ [37], that is, to better serve the needs of policy-makers by, for example, considering the wider political and local contexts in which policies are developed [38]. However, to date, no study has empirically investigated what features of HCP training evaluation studies are judged to be important for decision-making by policy-makers in LMICs, nor the extent to which evaluations based on the widely used Kirkpatrick model align with the evidence needs of policy-makers for resource allocation decisions.

This study aims to understand the factors that policy-makers consider important in evaluation studies to inform decisions on investments in HCP training programmes. We use China as a case study where resource allocation decisions about potential scale-up (using domestic funding) are being made about an externally funded pilot TB HCP training programme. Specifically, we investigate the extent to which evaluations based on the Kirkpatrick model meet the information needs of Chinese policy-makers and develop an evaluation framework for the design of policy-relevant training programme evaluations.

## Methods

### Study setting and participants

In recognition of the need to provide improved TB care at peripheral health facilities in China [39], two key health policy bodies – the Centre for Disease Control (CDC) and the Chinese Medical Association (CMA) – have embarked on pilot training programmes on TB management for doctors, nurses and laboratory technicians in selected provinces. These pilot programmes have largely been supported by funding from external donors over the past decade. Decisions need to be made about whether further investments from national and provincial health budgets should take place to continue and scale-up the pilot training programmes, and evaluations to inform policy-makers involved in resource allocation decisions are therefore being designed and conducted.

We conducted a qualitative study with officials in China between February and June 2016. A purposive sampling method was used to recruit key informants involved in resource allocation decisions or technical advisory roles related to infectious disease control programmes. Participants were approached prior to the study by means of a conference call and online presentation about the study. A total of 30 participants were recruited in this study, including directors of provincial and national CDCs in China, high-level managers of the CMA, and senior staff at tertiary hospitals leading HCP training programmes (Table 1). All participants had experience in planning and management of HCP training interventions. None of the participants approached refused to participate.

**Table 1:** Participant characteristics

	Interviews	Focus group discussions
Total participants	10	20
Female (%)	3 (30%)	13 (65%)
Organisation		
Centre for Disease Control representatives	4 (40%)	16 (80%)
Chinese Medical Association representatives	3 (30%)	4 (20%)
Hospital managers	3 (30%)	0
Geographical scope of work		
National level	6 (60%)	11 (55%)
Provincial level	4 (40%)	9 (45%)

### Data collection and analysis

We conducted ten face-to-face, in-depth interviews (IDIs) and two focus group discussions (FGDs) consisting of ten participants each. Open ended questions about how officials make decisions on investments in new disease control programmes were asked in the IDIs; the question phrasing was developed by a native Chinese researcher and pilot tested on Chinese doctors (not part of the study) to check that questions were clearly and appropriately articulated. The main topics covered included limitations of current training evaluation approaches, information needed to determine if a training programme is successful, factors policy-makers consider when presented with an evaluation report, and how policy-makers weigh different sources of information. A participatory exercise involving discussion of alternative evaluation approaches was used to initiate FGDs and to encourage exchange of views between participants [40]. Brief information (summarised in Box 1) was presented to participants on a series of slides before they started the FGDs.

## Box 1. Summary of hypothetical evaluation designs presented to officials and discussed in terms of importance of information provided for decision-making during the FGDs

**Table:** 

• Knowledge assessment: All trained healthcare providers (HCPs) were asked to complete three structured questionnaires at the start of the training, immediately after the training and 6 months after the training. Scores from the pre-training test were compared to the scores from the first and second post-training tests.
• Practical assessment: Standardised patients who were trained to present with TB symptoms visited selected trained HCPs in their health facilities. The medical practice of trainees was assessed by standardised patients on a scale of 1–10.
• Cost-effectiveness projection: The total cost of the HCP training programme and estimated improvement in patient level outcomes were calculated and compared.

IDIs and FGDs were led by a native female Chinese researcher trained in qualitative research methods as part of an ongoing PhD programme (SW), and were audio-recorded. Additional field notes were taken by a note-taker. All data was collected in a neutral location (hotel meeting room) during an annual conference attended by the participants. After data collection, audio recordings were transcribed verbatim in Chinese and translated into English by the Chinese researcher (SW). Participants were de-identified and numbered in the transcripts.

We conducted a thematic analysis – involving a search for themes that emerge as being important to the description of the phenomenon – employing an interpretive approach in which identified themes are supported by excerpts from the raw data to ensure that data interpretation remains directly linked to the words of the participants [41]. In order to organise the data to identify and develop themes from them, we coded each transcript line by line. Our coding process involved recognising an important moment in the responses and encoding it (seeing it as something) in advance of interpretation [42]. Our analysis started with a deductive coding phase followed by an inductive coding phase [43]. During the deductive coding phase, translated transcripts were organised and coded line by line manually using a coding frame developed a priori based on the Kirkpatrick model’s four components of reaction, learning, behaviour and results (Table 2). Data that did not fit into the four Kirkpatrick model components were then coded inductively, allowing themes to emerge directly from the data, by two researchers in parallel (SW and MK). Initial categories of coding emerging during the inductive coding phase were compared with subsequent coding and refined until all the data were sorted in line with the constant comparison technique [44]. Codes were then compared between researchers and collated into potential subthemes and themes using an iterative consensus decision-making process. Reporting followed consolidated criteria for reporting qualitative research (COREQ) [45].

**Table 2:** The four levels of the Kirkpatrick model and their definitions

Outcome level	Definition
Reaction	Assess how training participants react to the training and their perceived value of the training.
Learning	To what degree participants acquire intended knowledge, skills and attitudes based on participation in the learning event.
Behaviour	To what degree participants apply what they learned at training sessions on the job.
Programmatic results	Measure of the improvements that are expected in the team, programme or other context in which the trainee works. For example, successful treatment rate, case detection rate or patient satisfaction with services delivered by trained HCPs.

### Ethical approval

The research was approved by the Ethics Committee of the London School of Hygiene and Tropical Medicine and the National University of Singapore. We also received approvals from the China CDC and CMA representatives prior to conducting the study. Each interviewee was provided a consent form summarising objectives and methods of the research and highlighting the confidentiality and anonymity of interviewees’ responses. All interviewees read the information sheet and signed the consent form.

## Results

Our analysis identifies a number of features of HCP training evaluation studies that policy-makers judged to be important for informing decision-making surrounding resource allocation and training programmes. Informants indicated that the inclusion of information related to the direct outcomes of the training programme, as captured in the Kirkpatrick model, was essential. We also identified additional factors that contribute to the translation of evaluation study results into policy, which are not captured in evaluations designed solely using the Kirkpatrick model. We first summarise our findings and then propose a framework that captures a wider range of factors that are perceived to be important by policy-makers when considering evidence from training programme evaluation studies.

### Information needed by health policy-makers that is captured by the Kirkpatrick model

#### Reaction

In line with the Kirkpatrick model, almost all officials agreed that reaction – a measure of satisfaction of trainees with respect to the training programme – was an important component in training evaluation.

“*I think if a HCP training programme is successful, it should be determined by the HCPs, if they are satisfied with the training programme and its effectiveness.*” – IDI, national policy-maker

#### Learning

In addition to the reaction of trainees, officials acknowledged the importance of knowledge gain as one of the fundamental indicators of training effectiveness. It was also emphasised that, as illustrated by the quote below, evidence of both short- and long-term change in knowledge was important, and concerns were raised about, what informants perceived to be, limited evidence on long-term knowledge retention.

“*… I will think about the short-term change and also the long-term change including after training at the knowledge level how much has changed.*” – IDI, national policy-maker

#### Behaviour

Despite consensus among officials that learning was an essential component of any training evaluation, the majority emphasised that knowledge gain alone was not enough to determine the effectiveness of training programmes. Behaviour change of trainees in line with the training programme content was considered critical, but at the same time, the most difficult component to measure objectively. Our analysis suggests that the second and third components of the Kirkpatrick model were linked as far as officials were concerned. This view was held by officials working in national bodies such as the CDC and CMA as well as clinical experts.

“*Behaviour change is one goal. The first level* [knowledge gain] *is fundamental. But it is not enough to only gain knowledge. After gaining knowledge, you need behaviour change.*” – FGD Group A

“*I think, from a clinical perspective, effectiveness means that the performance of doctors is improved and standardised. But how to evaluate its effectiveness, how to assess if the job performance has improved, it is very hard to do.*” – FGD Group B

#### Programmatic results

Finally, in relation to the fourth component of the Kirkpatrick model – programmatic results – one official (IDI, hospital director) recognised that successful HCP training programmes would eventually have a positive influence on patient-level outcomes and overall disease control. However, whether to include the impact on patient-level outcomes as an indicator of the success of training programmes was debated among officials since they felt that impact on programmatic outcomes, such as incidence and treatment success rate, could be influenced by factors other than the training programme.

“*For the training programme, if you look at the impact, the best data is how many patients get good service or how much decline of prevalence or incidence. That one is an impact indicator. It is good, but this kind of indicator sometimes has mixed reasons. It is not only training to make the change.*” – IDI, national policy-maker

### Additional factors considered by policy-makers that are not direct training programme outcomes

While officials commented on the importance of the four components of the Kirkpatrick model, our analysis also found that the Kirkpatrick model on its own may not be sufficient to meet the information needs of policy-makers. As such, we identified six additional factors that were judged to be important for decision-making about investments in training.

#### Broader or indirect programmatic results of the training programme

In addition to expected direct results related to intended goals of the training interventions, we found that policy-makers consider indirect, wider benefits of the training. For example, an expanded pool of experienced trainers to lead on capacity building for other diseases, or experiences and lessons learned in management from implementation of the training programme were put forward as wider aspects that are important to assess, particularly from the perspective of officials working in national health policy bodies such as the CDC and CMA.

“*There are some targets that we did not set when we were designing the programme, but we are able to accomplish them…In the training programme, we also trained some trainers and teachers. After the programme is over, they can keep doing their job and train other doctors. And how we can reflect this in the evaluation is also very important.*” – IDI, hospital director

During the FGDs when hypothetical evaluation designs were discussed, there was consensus that it is challenging but important to consider the wider or indirect outcomes of training, particularly when evaluating the cost-effectiveness; there was a common feeling that effectiveness can be defined too narrowly, which is problematic from the perspective of officials.

#### Resources required

Although not included in the Kirkpatrick model, the cost of a training programme, in terms of both direct and indirect resources required, was considered an important component of training evaluation by officials. Direct resources that officials identified for assessing as part of an evaluation included costs for transportation and accommodation of trainees, trainers’ salaries and cost of training facilities; indirect resources that were not directly measurable in monetary terms included input required from various groups of staff and increased workload. Our analysis indicated that having enough resources available in the long term to cover the essential components of the training programme was a key concern of officials, and that evaluations which do not provide such information may, therefore, be of limited importance in informing decisions.

“*We will definitely consider the cost for training. For example, the cost of transportation and accommodation for trainees, and the remuneration for teachers… Then the local hospitals will not provide funding for their doctors and nurses to participate training programmes. If the doctors are asked to pay for the training, they definitely are not willing to participate.*” – IDI, hospital director

#### Sustainability

The third component discussed by officials but not captured in the Kirkpatrick model is sustainability, which was defined by participants as the potential to run the training programme effectively for several years. While one interviewee, who was a senior clinician (IDI, hospital director), suggested that he would not know how to assess sustainability from the information present in an evaluation report, other officials agreed on the importance of judging the sustainability from a policy-setting perspective based on research evidence. Specifically, officials expressed their need for information on the contextual factors of a programme that are important for determining long-term continuation. This included whether there is local support and demand from communities and commitment from partner organisations (regional health facilities) involved in implementation to continue the training programme. Another key factor mentioned as part of discussions on sustainability during IDIs was whether costs involved in running the training programme would be met by funders willing to continue investment (IDI hospital director in Beijing, national policy-maker, hospital director in Harbin). Here, a useful evaluation could present information about costs and resources required to continue or scale-up the training programme, but evaluators may not be in a position to assess future funding commitments. In addition, the usefulness of information about the cost-effectiveness of training programmes in relation to assessments of sustainability and willingness of funders to continue investing was discussed in both FGDs; here, the need for this information was widely considered important but there were mixed views about how effectiveness should be defined and whether policy-makers would be able to interpret results of cost-effectiveness analyses appropriately to inform decisions.

“*And it* [cost-effective analysis] *is definitely needed. If you don’t do it, you can hardly determine if we are going to invest in the future. So the main problem is that what indicators to use as an output* [for effectiveness]*, which is most difficult. But we have to do this analysis. If we don’t do this, it will be hard to evaluate the programme as a whole in the future.*” – FGD A

“*The quantification of cost-effectiveness is very important, but cost-effectiveness analysis is not a popular research area* [in China]… *there are still many problems concerning the design of indicators and calculation methods. Therefore, this is very important. But how to utilise these* [cost-effectiveness] *studies, how to make better use of those realistic indicators and information collected, those are the objectives we* [policy-makers] *need to achieve.*” – FGD A

While both FGDs indicated that the selection of appropriate indicators for a cost-effectiveness analysis is important in providing policy-makers with an assessment of sustainability, there was no conclusion on what the optimal indicators of effectiveness would be. Defining effectiveness too narrowly, as discussed earlier, was a concern highlighted during FGDs with respect to some previous evaluations seen by respondents.

A third element that was considered important in assessment of sustainability was the level of political support to make sufficient resources available to continue the programme. In addition to information on whether the programme had met its goals, interviewees put forward a range of different factors that influence political support, which are often outside the scope of typical evaluation studies. This included information on whether the disease area covered by the training programme – in this case TB – is considered a priority area for investment in light of competing priorities (IDI hospital director), and whether human resource capacity building was part of the country’s overall strategy (IDI national policy-maker).

“*Other infectious diseases like HIV or hepatitis B are related to individual behaviour, for example, hepatitis A is resulted from unclean food. HIV is a result of behaviour; if we can regulate our behaviour, we can control the transmission of HIV. But TB is different. It can infect you when you breathe. So infected patients are very innocent, because the infection is not related to your life style or your behaviour. So that’s why I think TB is the disease that needs investment the most… In terms of if the programme can continue, there are a lot of factors, such as the willingness of collaborators, the effectiveness of the programme, and if the programme fits in the political environment, and the sustainability. If the programme is very good, but not sustainable, then it is meaningless.*” – IDI, C1

#### Scalability

Officials were also interested in the scalability of a training programme to other settings within the country. To determine if a training programme could be scaled up to other settings, officials expressed a need for information on changes that would be required to the original training programmes to adapt them for other areas. They were conscious of regional differences in economic or cultural factors and indicated that they would find information about whether a pilot programme successful in one setting would be easily applicable in another setting highly beneficial in an evaluation report.

“*We need this programme to promote the development of a standardised training programme so that it can be replicated in other provinces. We need to know if the programme is applicable to other settings. If this programme targets the issues in only one or two provinces, then it is not worth scaling-up.*” – FGD B

Officials also emphasised that they consider the availability of sufficient resources – financial and human – within different regions to cover a larger population and if a feasible scale-up plan was in place. Here, evaluators can provide information about resources required but an assessment of resource availability in regions for future expansion may not be within the scope.

#### Evaluation methodology

In addition to wanting evaluations to contain information about training outcomes, costs, sustainability and scalability, all officials we interviewed (who had high levels of technical training and analytical skills owing to their senior positions) indicated that they paid attention to the evaluation methodology applied; our analysis suggests that this has a large influence on their perception of the quality of the findings. Specifically, we found that officials were interested in the study design, including whether both short- and long-term effects of training were evaluated, whether the sample was representative and large enough to draw conclusions, and whether the evaluation took a before–after comparison approach or parallel control approach. During FGDs, officials agreed that there was no ‘gold standard’ or best approach to evaluate a training programme, but five diverse respondents emphasised the importance of the objectivity of outcome indicators and the need to report potential confounders and assess biases in evaluation report.

“*What I want to see from the evaluation report is an objective assessment of our programme, including the quality of implementation, and effectiveness. The most important thing is that it can objectively evaluate the implementation of this program.*” – IDI, hospital director

Elaborating on some of the weaknesses they observe in evaluation approaches, they explained that pre- and post-training to assess knowledge retention of trainees, which they are commonly presented with, does not provide adequate evidence on behaviour change or long-term knowledge retention, which is important to them.

“*That doesn’t mean just because they have knowledge today, they know it next month.*” – IDI, national policy-maker

#### Composition of evaluation team

Finally, we found that it was not only the outcomes assessed in training evaluations but also who is providing the information that mattered to informants. Factors related to the composition of the evaluation team influenced the perceived reliability and relevance of the evaluation results; these included the qualification of evaluators, the reputation of their institution in China and overseas, their perceived independence from the training programme, and their knowledge of local context. Specifically, our study indicated that Chinese officials put different weighting on information provided by local (Chinese) and foreign (non-Chinese) evaluators. Analysis of the FGDs indicated a widely held perception that, although Chinese evaluators might be familiar with local culture, language and system, officials feared that the close relations between local evaluators with stakeholders of the training programmes would cause bias in assessment and influence the accountability of evaluation results. Compared to local evaluators, most officials believed that foreign evaluators that are external to the institution could conduct more objective evaluations since they held no conflict of interest.

“*We trust evaluation conducted by independent third parties, because it’s more objective and there is no interest involved*” – IDI, hospital director

In addition, specific respondents highlighted that the reputation and international impact of foreign evaluators would raise the credibility of the evaluation results (FGD Group A). However, some officials were concerned about the fact that cultural or language differences between foreign evaluators and the locals would delay the evaluation activities and impact the evaluation results. Therefore, during FGDs, agreement was reached that a mixed team of local and foreign evaluators would be ideal from the perspective of officials.

“*I think it will be better if local and international institutions can collaborate.*” – FGD B

### A training evaluation framework centred on policy-makers’ needs

**Fig. 1:**
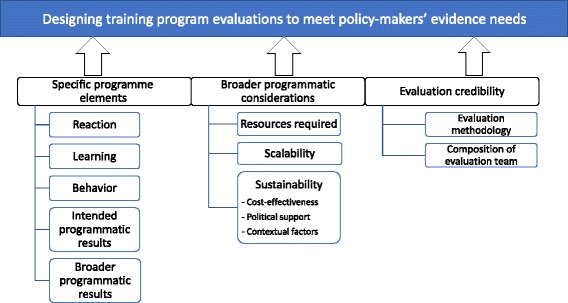
Modified training evaluation framework

Our qualitative study found that officials perceived the four components of the Kirkpatrick model to provide some policy-relevant information on specific programmatic elements of evaluations. However, our findings identified six additional factors that were judged to be important by policy-makers, suggesting that the Kirkpatrick model on its own may not be sufficient for meeting the evidence needs of policy-makers. Drawing on the ‘Interactive Model’ of policy-making outlined above, evidence can be more ‘fit for purpose’ if a broader range of factors, such as political context, are considered in evaluation studies [37]. As such, we propose a framework that incorporates, but moves beyond, the Kirkpatrick model to guide ‘policy relevant’ evaluations of training programmes (Fig. 1).

In this framework, three elements contribute to the policy relevance of a training evaluation, namely specific programme elements, broader programmatic considerations and evaluation credibility. The assessment of outcomes of training programmes, captured in the first element of the proposed framework, is linked to the four outcome levels from the Kirkpatrick model – reaction, learning, behaviour and results. Unlike the Kirkpatrick model, we breakdown ‘results’ into two categories in order to distinguish between intended direct results of the training and broader indirect programmatic results such as capacity-building of trainers that can be used in other training programmes.

We expand on the Kirkpatrick model by adding two new elements that are critical to policy-makers. The first is termed ‘broader programmatic considerations’, which includes direct and indirect resources required, sustainability of continued cycles of training over several years and scalability to other settings within the country. The second additional element added to the framework – termed ‘evaluation credibility’ – was based on a key finding of the study that policy-makers consider not only the information presented, but also who conducted the evaluation and the methodology. In terms of evaluation methodology, the evaluation study design, outcome indicators selected, and discussion of confounders and limitations of the evaluation approach were given importance by officials. In terms of who conducted the evaluation, objectivity and local knowledge were critical. Table 3 lists the definitions of all proposed components and provides examples of information needed to include.

**Table 3:** Definition of additional components and examples of information needed

Additional elements in proposed framework	Definition	Example of information needed
Broader programmatic results (Specific programme elements)	Indirect benefits from the training programmes	Enlarged pool of trainers; lessons learned from management of training programmes
Resources required (Broader programmatic considerations)	Resources invested in the training programme, including both direct and indirect costs	Human resource time devoted; trainers’ salary; cost for trainees’ accommodation
Sustainability (Broader programmatic considerations)	Whether the training programme can continue in the future	Contextual factors (demand from stakeholders to continue training); political support from local or national government; sufficient resources and funding
Scalability (Broader programmatic considerations)	Whether the training programme can be scaled up in other regions to cover a larger population	Local needs for the same training programme in other regions; ease of adaptability to different contexts; feasible plans for scale-up in place
Evaluation methodology (Credibility of evaluation)	Robustness of evaluation design and level of details provided to help policy-makers determine if objective approaches are used by evaluators	Study methodology including control groups; confounders and biases acknowledged
Composition of evaluation team (Credibility of evaluation)	Qualification of evaluators, their perceived independence and their knowledge of local context. The reputation of institutions to which the evaluation team members are affiliated also plays a role.	Potential conflicts of interest of evaluators; reputation of evaluators’ institution; technical background of evaluators; local language proficiency; experience in the local context

## Discussion

The importance of considering perceptions and information needs of policy-makers, and recognising their role as recipients or ‘receptors’ of research, is now solidly established [13]. Our qualitative analysis focused on the features of evaluation studies that policy-makers perceived to be important for informing resource allocation decisions about HCP training or capacity-building interventions. We aimed to address an important gap in information for researchers and funding organisations planning such evaluations. Indeed, considering the rapid increase in investments in HCP training, and the danger highlighted by WHO that “*poor training is a waste of resources*”, we sought to provide a guide for training evaluations based on the Kirkpatrick model [21]. The guide recognises the complexities of the policy-evidence nexus and the associated limitations of evaluation studies that are based solely on the Kirkpatrick model. Our analysis is the first to identify specific factors not captured in the Kirkpatrick model that are critical for policy-makers when making investment decisions based on evaluations of HCP training. The framework broadly focuses on the translation of programme evaluation to policy, rather than solely on the effectiveness of training programmes as captured by the Kirkpatrick model, in order to aid in the design and implementation of policy-relevant HCP training evaluations in LMIC contexts.

Consistent with the Kirkpatrick model, officials agreed that the reaction, knowledge (with an emphasis on long-term retention) and behaviour change of trainees were fundamental outcome indicators of the effectiveness of a training programme. There were mixed views on the relevance of programmatic outcome indicators, such as treatment success rates, since these would be influenced by factors other than HCP training. However, it was clear that evaluations based solely on the four levels of the Kirkpatrick model did not provide sufficient information for policy-makers to make decisions on future training programmes. We found that additional information on the inputs and costs, wider or indirect impacts of training, sustainability and scalability of training programmes to other parts of the country, are important to policy-makers and should therefore be reflected in evaluations. A major finding was that policy-makers do not only consider the information covered in evaluation studies, but also pay close attention to the design of evaluations and qualifications of those who conducted the evaluation; these factors were found to influence perceptions of the reliability of the results and are consistent with findings from studies on translation of research to policy [6, 10]. Specifically, a clear recommendation was that a combination of local (Chinese) and foreign (non-Chinese) researchers was ideal from the perspective of officials in our study, since foreign evaluators were thought to have fewer conflicts of interest and Chinese evaluators were familiar with local culture, language and systems.

### Strengths and limitations of proposed framework and the study methodology

Like other goal-based evaluation frameworks [29, 46–48], our proposed framework builds on the Kirkpatrick model, focusing on better addressing the evidence needs of policy-makers for decision-making. The elements identified by officials and incorporated into our modified framework address a gap in current evaluation approaches, and applying this framework when planning evaluations may reduce the barriers to the translation of research evidence into policy [2]. For example, even though it is not commonly assessed among current evaluation studies [49], the cost of rolling out a training programme, and the likely availability of sufficient resources in the long-term, is an important consideration of policy-makers, which has also been found in other studies [2, 50]. Policy-makers are aware that it is counterproductive when funds fall short before the programme achieves its intended goals and after significant start-up human and fiscal resources have been invested [51], and therefore including information on sustainability is essential for policy decisions.

In line with previous studies, we found that perception of the quality of the research and research team is a major factor influencing the use of research results [8, 10]; our framework explicitly includes this important element which helps to capture the complexity of researcher and policy-maker interactions in evidence-based policy settings [52]. Furthermore, our findings indicate that policy-makers are not only concerned with the internal validity of the evaluation, but also external validity in terms of whether the evaluation results demonstrate scalability [53].

While we largely found consistent views across a range of officials working in different organisations and provinces in China, we acknowledge that we focused on a relatively small group of influential stakeholders that were working in infectious disease control, and that Chinese officials working on non-communicable diseases may have differing perspectives. We also recognise that policy-makers in other countries may differ in their considerations when making decisions on training programme investments. In particular, we found that the officials interviewed as part of this study were highly knowledgeable about evaluation study designs, which may have influenced their views on evaluation teams and methodologies; to assess a broader applicability of the framework, it could be tested in other LMIC settings and with Chinese stakeholders working outside of infectious disease control. We also recognise specific limitations of FGDs, in which participants may be influenced by ‘dominant voices’ to agree on a ‘group opinion’ [54]. To enhance the quality of data collected we used an exercise to initiate the FGDs that enabled participants to share their reactions to a set of hypothetical evaluation designs one by one, and had a skilled native researcher facilitate the FGDs. Comparing responses across FGDs and IDIs, we noticed that some subjective themes related to sustainability (including political commitment) and scalability (including regional differences in capacity) were discussed more openly in IDIs. However, FGDs were effective in generating a lively debate about the composition of an ideal evaluation team; IDIs generated less rich responses about this question.

Our study was conducted with a focus on how training evaluations can be designed to better inform policy decisions, and an additional aspect, which is beyond the scope of this study, is the skills of policy-makers in being able to interpret evaluation results effectively [2]. Finally, in terms of the research team’s reflexivity, we acknowledge that our focus on health policy and systems research encouraged us, in advance, to question the simplicity of the Kirkpatrick model and look for wider factors that influence policy-makers when considering evidence presented in evaluation studies, as we believe that the policy process is complex [4]. We acknowledge that this study focused specifically on factors influencing the use of evidence in evaluation studies by policy-makers, and emphasise that research evidence is only one of several drivers of policy decisions [4, 52, 55].

## Conclusions

In light of the large investments in training to address a severe need for skilled human resources for health in LMICs, evaluations to inform policy-makers about future investments in training are critical. We found that evaluations focusing narrowly on direct training outcomes, as captured by the Kirkpatrick model, do not address several factors that are important to policy-makers. Six factors that policy-makers judged to be important for policy-relevant evaluation studies included broader indirect outcomes of the training programme, direct and indirect resources required, sustainability, scalability, evaluation methodology and composition of the evaluation team. Based on these findings, we have developed an evidence-based framework, which includes but expands beyond the Kirkpatrick model, to provide conceptual and practical guidance that aids in the design of training programme evaluations that are suited to meet the evidence needs of policy-makers and to inform policy decisions.

## Abbreviations

CDC: Center for Disease Control and Prevention;
CMA: Chinese Medical Association
COREQ: Consolidated Criteria for Reporting Qualitative Research
FGD: focus group discussion
HCP: healthcare provider
IDI: in-depth interview
LMIC: low- and middle-income countries
TB: tuberculosis

## References

[1] Lindblom C, Cohen D (1979). Usable Knowledge: Social Science and Social Problem Solving.

[2] Hyder AA, Corluka A, Winch PJ, El-Shinnawy A, Ghassany H, Malekafzali H, Lim MK, Mfutso-Bengo J, Segura E, Ghaffar A (2011). National policy-makers speak out: are researchers giving them what they need?. Health Policy Plan..

[3] Weiss C (1979). The many meanings of research utilization. Public Adm Rev..

[4] Hawkins B, Parkhurst J (2016). The ‘good governance' of evidence in health policy. Evid Policy..

[5] Aaserud M, Lewin S, Innvaer S (2005). Translating research into policy and practice in developing countries: a case study of magnesium sulphate for pre-eclampsia. BMC Health Serv Res..

[6] Albert M, Fretheim A, Maiga D (2007). Factors influencing the utilization of research findings by health policy-makers in a developing country: the selection of Mali’s essential medicines. Health Res Policy Syst..

[7] Hennik M, Stephenson R (2005). Using research to inform health policy: barriers and strategies in developing countries. J Health Commun..

[8] Trostle J, Bronfman M, Langer A (1999). How do researchers influence decision-makers? Case studies of Mexican policies. Health Policy Plan..

[9] Lavis JDH, Oxman A, Denis JL, Golden-Biddle K, Ferlie E (2005). Towards systematic reviews that inform health care management and policy-making. J Health Serv Res Policy..

[10] Innvaer S, Vist G, Trommald M, Oxman A (2002). Health policy-makers' perceptions of their use of evidence: a systematic review. J Health Serv Res Policy..

[11] Dobbins M, Ciliska D, Cockerill R, Barnsley J, DiCenso A (2002). A framework for the dissemination and utilization of research for health-care policy and practice. Online J Knowl Synth Nurs..

[12] Kouri D (1997). Introductory Module: Introduction to Decision Theory and Practice.

[13] Hanney S, Gonzalez-Block M, Buxton M, Kogan M (2003). The utilisation of health research in policy-making: concepts, examples and methods of assessment. Health Res Policy Syst..

[14] Schneider A, Ingram H (1997). Policy Design for Democracy.

[15] Beaglehole R, Dal Poz MR (2003). Public health workforce: challenges and policy issues. Hum Resour Health..

[16] World Health Organization (2006). The World Health Report 2006: Working Together For Health.

[17] Figueroa-Munoz J, Palmer K, Dal Poz M, Blanc L, Bergström K, Raviglione M (2005). The health workforce crisis in TB control: a report from high-burden countries. Hum Resour Health..

[18] Wu Q, Zhao L, Ye XC (2016). Shortage of healthcare professionals in China. BMJ..

[19] Bowser D, Sparkes SP, Mitchell A, Bossert TJ, Barnighausen T, Gedik G, Atun R (2014). Global Fund investments in human resources for health: innovation and missed opportunities for health systems strengthening. Health Policy Plan..

[20] Wu S, Roychowdhury I, Khan M (2017). Evaluations of training programs to improve human resource capacity for HIV, malaria and TB control: a systematic review of methods applied and outcomes assessed. Trop Med Health..

[21] World Health Organization (2010). Evaluating Training in WHO.

[22] Kirkpatrick D (2006). Evaluating Training Programs: The Four Levels (3rd edition).

[23] Phillips PPJ (2001). Symposium on the evaluation of training. Int J Train Dev..

[24] Kraiger KFJ, Salas E (1993). Application of cognitive, skill-based, and affective theories of learning outcomes to new methods of training evaluation. J Appl Psychol..

[25] Arthur WBW, Edens P, Bell S (2003). Effectiveness of training in organizations: a meta-analysis of design and evaluation features. J Appl Psychol..

[26] Guskey T. Five Levels of Professional Development Evaluation: North Central Regional Educational Laboratory (NCREL); 2002.

[27] Kaufman R, Keller J, Watkins R (1996). What works and what doesn't: evaluation beyond kirkpatrick. Perform Improv..

[28] Kearns P, Miller T (1997). Measuring the Impact of Training and Development on the Bottom Line.

[29] Hamblin AC (1974). Evaluation and Control of Training.

[30] Brauchle P, Schmidt K (2004). Contemporary approaches for assessing outcomes on training, education, and HRD programs. J Ind Teach Educ..

[31] O'Malley G, Perdue T, Petracca F (2013). A framework for outcome-level evaluation of in-service training of health care workers. Hum Resour Health..

[32] Alvarez K, Salas E, Garofano C (2004). An integrated model of training evaluation and effectiveness. Hum Resour Dev Rev..

[33] Bates R (2004). A critical analysis of evaluation practice: the Kirkpatrick model and the principle of beneficence. Eval Program Planning..

[34] Naude CE, Zani B, Ongolo-Zogo P, Wiysonge CS, Dudley L, Kredo T, Garner P, Young T (2015). Research evidence and policy: qualitative study in selected provinces in South Africa and Cameroon. Implement Sci..

[35] Sackett PR, Mullen EJ (1993). Beyond formal experimental design: towards an expanded view of the training evaluation process. Pers Psychol..

[36] Bowen S, Zwi AB (2005). Pathways to “evidence-informed” policy and practice: a framework for action. PLoS Med..

[37] Hunter DJ (2009). Relationship between evidence and policy: a case of evidence-based policy or policy-based evidence?. Public Health..

[38] Leir S, Parkhurst J. What is Good Evidence for Policy? London: London School of Hygiene and Tropical. Medicine. 2016;

[39] Hutchison C, Khan MS, Yoong J, Lin X, Coker RJ (2017). Financial barriers and coping strategies: a qualitative study of accessing multidrug-resistant tuberculosis and tuberculosis care in Yunnan, China. BMC Public Health..

[40] Ritchie J, Lewis J, Nicholls CM, Ormston R (2013). Qualitative Research Practice: A Guide for Social Science Students and Researchers.

[41] Rice P, Ezzy D (1999). Qualitative Research Methods: A Health Focus.

[42] Boyatzis R (1998). Transforming Qualitative Information: Thematic Analysis and Code Development.

[43] Saldana J (2009). The Coding Manual for Qualitative Researchers.

[44] Glaser B, Strauss A (1967). The Discovery of Grounded Theory: Strategies for Qualitative Research.

[45] Tong A, Sainsbury P, Craig J (2007). Consolidated criteria for reporting qualitative research (COREQ): a 32-item checklist for interviews and focus groups. Int J Qual Health Care..

[46] Bramley P (1996). Evaluating Training Effectiveness.

[47] Warr P, Bird M, Rackcam N (1978). Evaluation of Management Training.

[48] Foxon M (1989). Evaluation of training and development programs: a review of the literature. Aust J Educ Technol..

[49] Wu S, Roychowdhury I, Khan M (2017). Evaluating the impact of healthcare provider training to improve tuberculosis management: a systematic review of methods and outcome indicators used. Int J Infect Dis..

[50] Johns B, Baltussen R, Hutubessy R (2003). Programme costs in the economic evaluation of health interventions. Cost Eff Resour Alloc..

[51] Shediac-Rizkallah MC, Bone LR (1998). Planning for the sustainability of community-based health programs: conceptual frameworks and future directions for research, practice and policy. Health Educ Res..

[52] Hyder AA, Bloom G, Leach M, Syed SB, Peters DH (2007). Future Health Systems: Innovations for Equity. Exploring health systems research and its influence on policy processes in low income countries. BMC Public Health..

[53] Onwuegbuzie AJ. Expanding the Framework of Internal and External Validity in Quantitative Research**.** 2000. https://eric.ed.gov/?id=ED448205. Accessed 12 Feb 2018.

[54] Smithson J (2000). Using and analysing focus groups: limitations and possibilities. Int J Social Research Methodology..

[55] Black N (2001). Evidence based policy: proceed with care. BMJ..

